# *In Vivo* Non-radioactive Assessment of mGlu5 Receptor-Activated Polyphosphoinositide Hydrolysis in Response to Systemic Administration of a Positive Allosteric Modulator

**DOI:** 10.3389/fphar.2018.00804

**Published:** 2018-07-31

**Authors:** Anna R. Zuena, Luisa Iacovelli, Rosamaria Orlando, Luisa Di Menna, Paola Casolini, Giovanni Sebastiano Alemà, Gabriele Di Cicco, Giuseppe Battaglia, Ferdinando Nicoletti

**Affiliations:** ^1^Department of Physiology and Pharmacology “Vittorio Erspamer,” Sapienza University, Rome, Italy; ^2^IRCCS Neuromed, Pozzilli, Italy

**Keywords:** mGlu5 receptors, receptor signaling, polyphosphoinositide hydrolysis, InsP levels, VU0360172, MTEP

## Abstract

mGlu5 receptor-mediated polyphosphoinositide (PI) hydrolysis is classically measured by determining the amount of radioactivity incorporated in inositolmonophosphate (InsP) after labeling of membrane phospholipids with radioactive inositol. Although this method is historically linked to the study of mGlu receptors, it is inappropriate for the assessment of mGlu5 receptor signaling *in vivo*. Using a new ELISA kit we showed that systemic treatment with the selective positive allosteric modulator (PAM) of mGlu5 receptors VU0360172 enhanced InsP formation in different brain regions of CD1 or C57Black mice. The action of VU0360172 was sensitive to the mGlu5 receptor, negative allosteric modulator (NAM), MTEP, and was abolished in mice lacking mGlu5 receptors. In addition, we could demonstrate that endogenous activation of mGlu5 receptors largely accounted for the basal PI hydrolysis particularly in the prefrontal cortex. This method offers opportunity for investigation of mGlu5 receptor signaling in physiology and pathology, and could be used for the functional screening of mGlu5 receptor PAMs in living animals.

## Introduction

mGlu5 metabotropic glutamate receptors are coupled to G_q/11_, and their activation stimulates phospholipase-C-mediated polyphosphoinositide (PI) hydrolysis with ensuing formation of inositol-1,4,5-trisphosphate (InsP_3_) and diacylglycerol (reviewed by Nicoletti et al., 2011). mGlu5 receptors play a key role in mechanisms of activity-dependent synaptic plasticity and are promising candidate drug targets for neuroprotective drugs (reviewed by Bruno et al., 2017). Selective ligands of mGlu5 receptors (both positive and negative allosteric modulators) are under development for the treatment of numerous CNS disorders, such as unipolar depression, obsessive-compulsive disorder, schizophrenia, autism, chronic pain, and Parkinson’s disease (Vinson and Conn, 2012; Nicoletti et al., 2015; Bruno et al., 2017; Rutrick et al., 2017). This raises the interest on how expression and function of mGlu5 receptors change during CNS development (Minakami et al., 1995; Casabona et al., 1997), in association with learning and memory processes (Riedel et al., 2000), in response to stress (Zuena et al., 2008; Yim et al., 2012), and under pathological conditions. Abnormalities in the expression or function of mGlu5 receptors have been associated with schizophrenia (Ohnuma et al., 1998; Gupta et al., 2005; Matosin et al., 2013, 2015; Fatemi and Folsom, 2015; Lum et al., 2017; Zurawek et al., 2017), focal cortical dysplasia (DuBois et al., 2016), absence epilepsy (D’Amore et al., 2015), Down’s syndrome (Iyer et al., 2014), neuropathic pain (Lin et al., 2015), Fragile-X syndrome, and other types of monogenic autism (Huber et al., 2002; Bear et al., 2004; Giuffrida et al., 2005; Ronesi et al., 2012; Pignatelli et al., 2014).

Most of the studies investigating mGlu5 receptor-mediated PI hydrolysis have been performed in *ex vivo* systems, i.e., in brain slices preloaded with [^3^H]-*myo*-D-inositol and challenged with the mGlu1/5 receptor agonist, 3,5-dihydroxyphenylglycine (DHPG). Radioactive inositol is incorporated into inositol phospholipids, and stimulation of PI hydrolysis is routinely assessed by measuring the amount of radioactive inositol monophosphate (InsP, a degradation product of InsP_3_) after blocking its conversion into free inositol with lithium ions. Although this method is informative and is linked to the first descriptions of mGlu receptors (Sladeczek et al., 1985; Nicoletti et al., 1986), it incorporates a number of biases that preclude an accurate analysis of how mGlu5 receptors signal in physiological and pathological conditions. For example, (i) tissue from 4 to 10 rats or mice (depending on the brain region) must be pooled for slice preparation; (ii) the uncontrolled extracellular concentrations of endogenous glutamate in brain slice preparations renders this model inappropriate for the study of the action of different classes of positive allosteric modulators (PAMs) on native mGlu5 receptors; (iii) measurements of radioactive InsP are routinely performed by anion exchange chromatography followed by radioactivity counting with no information on endogenous InsP levels (and, therefore, on the specific activity of [^3^H]-InsP); and, (iv) when applied to *in vivo* studies the method requires the intracerebral injection of radioactive inositol.

Here, we demonstrate that it is possible to assess mGlu5 receptor-mediated PI hydrolysis in mice systemically injected with lithium ions followed by a selective mGlu5 receptor PAM by using the Cisbio IP-One ELISA kit, which detects endogenous InsP levels (Trinquet et al., 2011). This method may allow a functional analysis of mGlu5 receptor function in all brain regions of *individual* animals, and may be of great help for the study of the effects of selective ligands on native mGlu5 receptors in physiology and pathology.

## Materials and Methods

### Animals

We used adult CD1 or C57Black male mice (7–8 weeks of age) obtained from Charles River (Calco, Italy), and mGlu5 receptor knockout (mGlu5^-/-^) mice, obtained by heterozygous breeding (B6;129-Grm5tm1Rod/J, Stock No: 003558, originally purchased from Jackson Laboratory, Bar Harbor, ME, United States). All mice were individually genotyped for the mGlu5 receptor gene by polymerase chain reaction to identify wild-type, heterozygous and knockout mice. All mice were housed in a controlled-temperature room (21–23°C, humidity 40–50%) and maintained on a 12-h light/dark cycle with food and water *ad libitum*. All efforts were made to minimize the number of animals used and to alleviate their discomfort. All experimental procedures were performed in conformity with the Italian (D.L. 26/2014) and European Union Directive (2010/63/EU) on the protection of animals used for scientific purposes. Experiments were approved by the Italian Ministry of Health.

### Drugs and Treatments

Experiments were carried out in mice pretreated with lithium chloride. This pretreatment was expected to amplify receptor agonist-stimulated InsP formation owing to the ability of lithium to inhibit the conversion of InsP into free inositol (Berridge et al., 1982). Lithium chloride was purchased from Sigma-Aldrich (Milan, Italy), VU0360172 [*N*-cyclobutyl-6-(2-(3-fluorophenyl)ethynyl) pyridine-3-carboxamide] and MTEP 3-[(2-methyl-1,3-thiazol-4-yl)ethynyl]pyridine hydrochloride were purchased from Tocris Bioscience (Bristol, United Kingdom). Lithium chloride was dissolved in saline, VU0360172 and MTEP were dissolved in 10% Tween 80 and adjusted to pH 7.4 with NaOH. Drugs and vehicles were administered i.p. in a volume of 5 ml/kg body weight.

Doses used for VU0360172 and MTEP were selected on the basis of behavioral data in mice (Rodriguez et al., 2010; Hughes et al., 2013; Domin et al., 2014). Mice were treated with lithium chloride (2.5 or 10 mmol/kg, corresponding to 105 or 420 mg/kg) and, 1 h later, with VU0360172 (0.3, 3, or 30 mg/kg) and/or MTEP (10 mg/kg), or their vehicles. Two additional groups of CD1 mice were treated i.p. with either VU0360172 (30 mg/kg) or its vehicle without pretreatment with lithium chloride. One hour after drug injections, mice were killed by decapitation, and the prefrontal cortex, hippocampus, corpus striatum, hypothalamus, olfactory bulbs, and cerebellum were rapidly dissected and stored at -80°C until InsP measurements.

### Tissue Preparation and ELISA Measurement of InsP Levels

Frozen tissue was weighed and homogenized by sonication in 10 μl/mg of tissue of Tris-HCl buffer (100 mM; pH 7.5) containing 150 mM NaCl, 5 mM EDTA, 1% Triton X-100, 1% SDS. Homogenates were diluted 1:50 and InsP levels were assessed with the IP-One ELISA kit (Cisbio, Codolet, France) according to the manufacturer’s instructions. Mean intra-assay and inter-assay coefficients of variation were 4.68 and 4.83%, respectively.

### Immunoblotting

Western blot analysis of mGlu5 receptors was carried out in the hippocampus, prefrontal cortex, corpus striatum, olfactory bulb, hypothalamus, and cerebellum of four CD1 mice randomly selected from the groups used for the assessment of PI hydrolysis. An aliquot of tissue extract was added with protease inhibitors cocktail (Sigma-Aldrich, Cat. # 2714) and after protein determination, protein lysates (40 μg) were separated by SDS-PAGE electrophoresis and blotted onto nitrocellulose. The upper part of the membrane was probed with polyclonal anti-mGlu5 receptor antibody (Millipore, CA, United States, Cat. # AB5675, 1:3,000 dilution), and the lower part of the membrane was probed with monoclonal anti-β-actin antibody (Sigma-Aldrich, Cat. # A2228, 1:10,000 dilution). Immunoreactive bands were visualized by enhanced chemilumiscence (Westar, Nova 2011, Cyanagen, Bologna, Italy) using horseradish peroxidase-conjugated secondary antibodies. Densitometric analysis of the immunoreactive bands was performed by Image J (NIH, Bethesda, MD, United States).

## Results

### *In Vivo* Assessment of mGlu5 Receptor-Mediated PI Hydrolysis in Different Mouse Brain Regions

**FIGURE 1:**
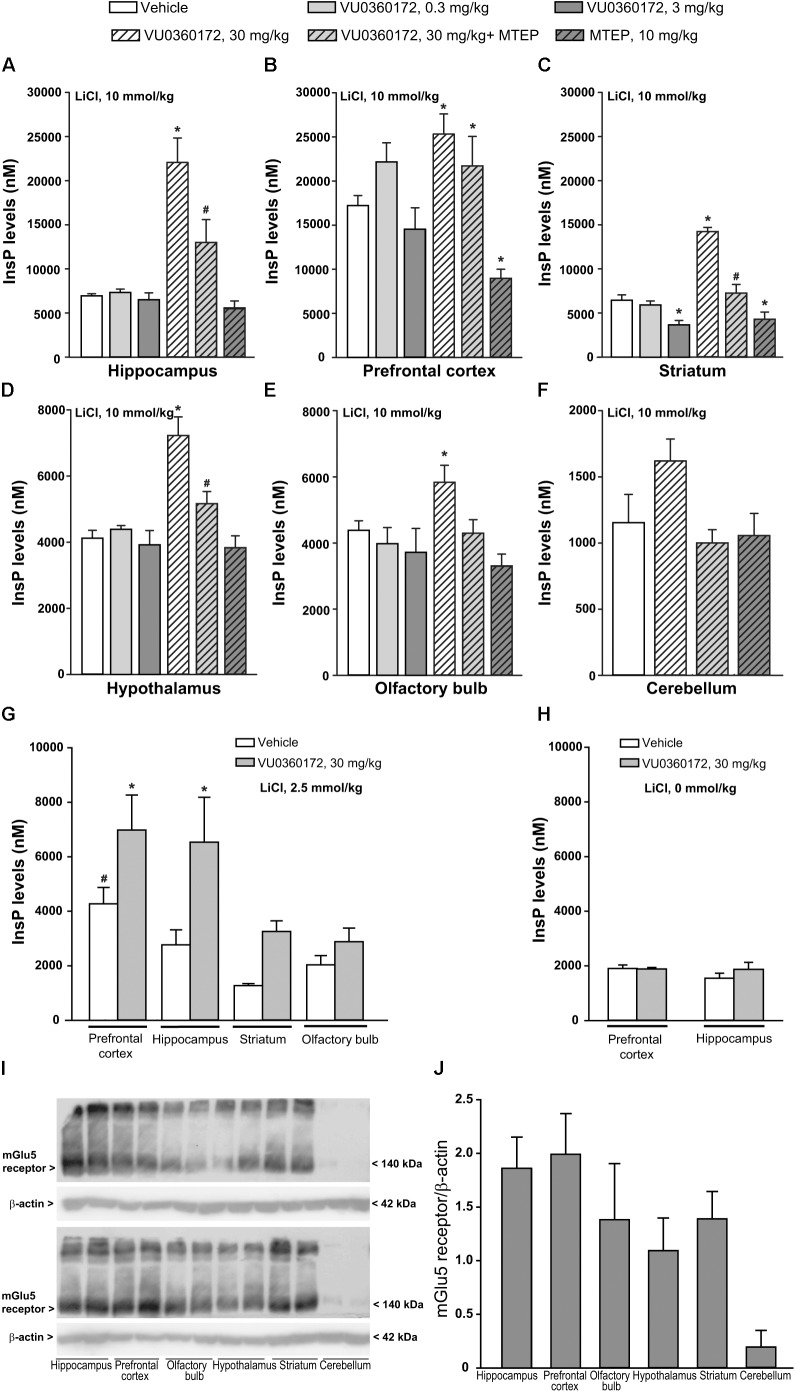
Measurements of endogenous InsP levels in CD1 mice treated with lithium ions and then challenged with the selective mGlu5 receptor PAM VU0360172. **(A–F)** InsP levels in selected brain regions of adult CD1 mice treated i.p. with 10 mmol/kg of lithium chloride (LiCl) followed by vehicle, VU0360172 (0.3, 3, or 30 mg/kg), MTEP (10 mg/kg), or VU0360172 (30 mg/kg) + MTEP. Values are means ± SEM of 5–12 mice per group. *P* < 0.05 vs. values obtained in mice treated with vehicle in the same brain region (^∗^) or vs. values obtained in mice treated with 30 mg/kg VU0360172 in the absence of MTEP (#) (One-way ANOVA + Fisher’s LSD; *F*_(5,38)_ = 19.82 for hippocampus; *F*_(5,38)_ = 7.33 for prefrontal cortex; *F*_(5,38)_ = 27.47 for striatum; *F*_(5,35)_ = 12.28 for hypothalamus; *F*_(5,35)_ = 3.28 for olfactory bulb). **(G)** InsP levels in selected brain regions of adult CD1 mice treated i.p. with 2.5 mmol/kg of LiCl followed by vehicle or VU0360172 (30 mg/kg). Values are means ± SEM of 5–7 mice per group. *p* < 0.05 vs. values obtained in mice treated with vehicle in the same brain region (^∗^) or vs. values in the striatum or olfactory bulb of mice treated with vehicle (#) [One-Way ANOVA + Fisher’s LSD; *F*_(7,26)_ = 6.3]. **(H)** InsP levels in the prefrontal cortex and hippocampus of adult CD1 mice injected with VU0360172 (30 mg/kg) or its vehicle without pretreatment with LiCl. Values are means + SEM of 3-4 mice per group. **(I,J)** Western blot analysis of mGlu5 receptors in four CD1 mice randomly selected from the same groups as in **(A–F)**. Densitometric values are means ± SEM.

To examine whether an enhanced formation of InsP could be detected after systemic treatment with a selective mGlu5 receptor PAM, we used six groups of CD1 mice treated with vehicle, VU0360172 (0.3, 3, or 30 mg/kg), MTEP (10 mg/kg), or VU0360172 (30 mg/kg) + MTEP, respectively. All mice received a single injection of LiCl (10 mmol/kg) 1 h prior to drug administrations to inhibit the conversion of InsP into free inositol. Measurements of endogenous InsP levels were carried out in tissue extracts prepared from the prefrontal cortex, hippocampus, corpus striatum, hypothalamus, olfactory bulbs, and cerebellum. Basal InsP levels (i.e., levels detected in mice treated with vehicle) were not homogeneous in the selected brain regions and were substantially higher in the prefrontal cortex than in all other regions (**Table 1**). This might reflect a high level of endogenous activation or a constitutive activity of mGlu5 receptors because InsP levels in the prefrontal cortex were halved by treatment with the mGlu5 receptor NAM, MTEP (**Figure 1B**).

**Table 1:** Basal levels of endogenous InsP in different brain regions of CD1 mice.

Brain regions	InsP levels (nM)
Hippocampus	6950 ± 244
Prefrontal cortex	17215 ± 1148^∗^
Striatum	6446 ± 601
Hypothalamus	4124 ± 238
Olfactory bulb	4349 ± 288
Cerebellum	1154 ± 213


Mice were treated i.p. with 10 mmol/kg of lithium chloride, followed, one hour later, by the vehicle of VU0360172. Mice were killed 1 hour after the vehicle (i.e., 2 hours after lithium chloride). Values are means ± S.E.M. of 5–12 mice per group. *p<0.05 vs. all other brain regions (One-way ANOVA + Fisher’s LSD; F_(5,57)_ = 73.39).

The highest dose of the mGlu5 receptor PAM, VU0360172 (30 mg/kg), enhanced InsP levels by >3 fold in the hippocampus **Figure 1A**, by about twofold in the striatum (**Figure 1C**), and to a lower extent in the other brain regions (**Figures 1D–F**). Stimulation was negligible in the cerebellum (**Figure 1F**). In the prefrontal cortex of CD1 mice, VU0360172 treatment caused a small, albeit significant, increase in InsP formation, perhaps because of the high basal noise (**Figure 1B**). At doses of 0.3 or 3 mg/kg, VU0360172 failed to induce detectable changes in endogenous InsP levels (**Figures 1A–E**).

Stimulation of PI hydrolysis by VU0360172 was largely reduced by MTEP in the hippocampus, striatum, hypothalamus, and olfactory bulb (**Figures 1A,C–E**). Unexpectedly, in the prefrontal cortex co-treatment with VU0360172 and MTEP raised InsP levels by >2 fold as compared to values obtained with MTEP alone (**Figure 1B**).

We then examined the effect of VU0360172 in CD1 mice by lowering the dose of LiCl from 10 to 2.5 mmol/kg (corresponding to 105 mg/kg). Basal InsP levels were reduced in all selected brain regions, particularly in the prefrontal cortex where basal levels were about 20% of those found with 10 mmol/kg (compare **Figures 1A–C,E** with **Figure 1G**). In mice treated with 2.5 mmol/kg of LiCl VU0360172 (30 mg/kg) stimulated InsP formation in the hippocampus, prefrontal cortex, and corpus striatum, but not in the olfactory bulb (**Figure 1G**). In the prefrontal cortex, the extent of stimulation was greater in mice treated with 2.5 mmol/kg than in mice treated with 10 mmol/kg (62 vs. 32%) because of a higher signal-to-noise ratio (**Figure 1G**). In an additional experiment, two groups of mice were treated with 30 mg/kg of VU0360172 or its vehicle without pre-treatment with LiCl. Basal InsP levels were further reduced in the prefrontal cortex and hippocampus, as compared to values obtained after pretreatment with 2.5 mmol/kg of LiCl (compare **Figure 1G** and **Figure 1H**). Treatment with VU0360172 failed to enhance InsP formation in mice that had not been pretreated with LiCl (**Figure 1H**). The dependence on lithium on basal and agonist-stimulated InsP accumulation was expected on the basis of previous results obtained in mice challenged with muscarinic cholinergic receptor agonists (Popiolek et al., 2016). In four CD1 mice randomly selected from those used for the assessment of PI hydrolysis we examined mGlu5 receptor protein levels. Immunoblots showed a band at about 140 kDa corresponding to receptor monomers, and a higher size molecular band corresponding to receptor dimers. mGlu5 receptor protein levels were higher in the hippocampus and prefrontal cortex, followed by corpus striatum, hypothalamus, and olfactory bulbs, although there was no significant difference among the various brain regions (**Figures 1I,J**). Protein levels were very low in the cerebellum, as expected (Abe et al., 1992) (**Figures 1I,J**).

**FIGURE 2:**
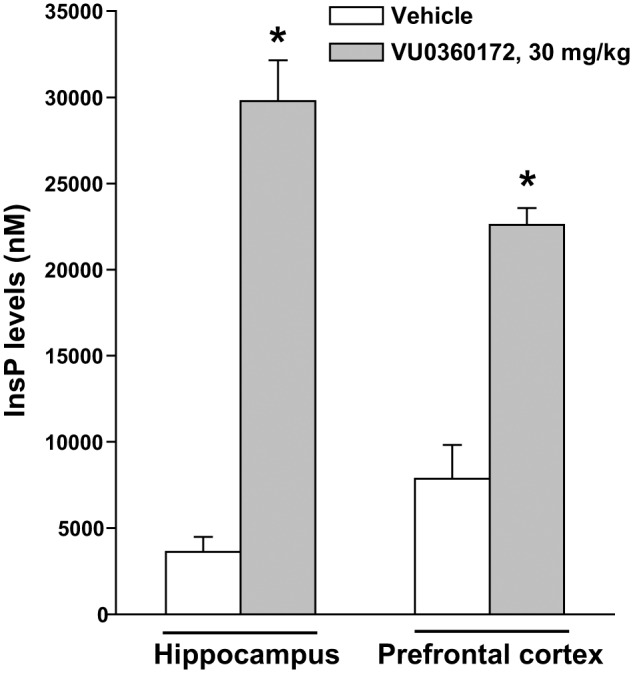
Assessment of mGlu5 receptor-mediated PI hydrolysis in the hippocampus and prefrontal cortex of C57Black mice challenged with VU0360172. Adult C57Black mice were treated i.p. with 10 mmol/kg of lithium chloride followed by vehicle or VU0360172 (30 mg/kg). Values are means ± SEM of six mice per group. ^∗^*p* < 0.05 vs. data obtained in mice treated with vehicle in the same brain region [One-Way ANOVA + Fisher’s LSD; *F*_(3,20)_ = 53.91].

To examine whether mGlu5 receptor-mediated PI hydrolysis *in vivo* was strain-dependent, we also treated C57Black mice with VU0360172 (30 mg/kg) and measured InsP levels in the hippocampus and prefrontal cortex. In the hippocampus of C57Black mice VU0360172 stimulated InsP formation by as much as sevenfold. In the prefrontal cortex, basal InsP levels were higher than in the hippocampus. However, treatment with VU0360172 enhanced InsP formation by almost threefold (**Figure 2**). Thus, the extent of mGlu5 receptor-mediated PI hydrolysis was greater in C57Black mice than in CD1 mice.

### VU0360172-Stimulated PI Hydrolysis *in Vivo* Was Abrogated in Mice With Genetic Deletion of mGlu5 Receptors

**FIGURE 3:**
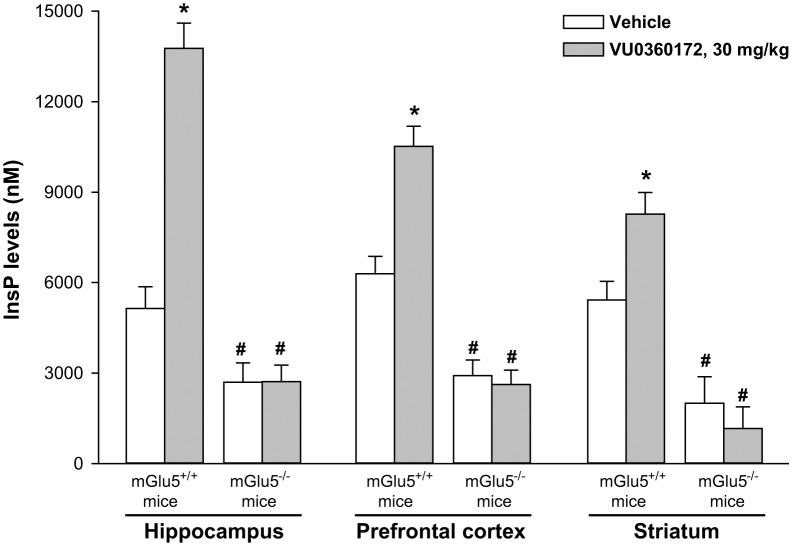
Systemic treatment with VU0360172 failed to stimulate PI hydrolysis in the hippocampus, prefrontal cortex and striatum of mice lacking mGlu5 receptors. mGlu5^+/+^ and mGlu5^-/-^ mice were treated with 10 mmol/kg of lithium chloride followed by either vehicle or VU0360172 (30 mg/kg). Values are means ± SEM of 3–6 mice for the prefrontal cortex, 3–7 mice for the hippocampus and 2–4 mice for the striatum *p* < 0.05 vs. the respective groups of vehicle-treated mice (^∗^) or vs. the respective groups of mGlu5^+/+^ mice (#) [Two-way ANOVA + Fisher’s LSD; *F*_(1,15)_ = 94.99 for hippocampus; *F*_(1,14)_ = 99.51 for prefrontal cortex; *F*_(1,8)_ = 51.15 for striatum].

To further demonstrate that VU0360172 was able to stimulate PI hydrolysis *in vivo* through the activation of mGlu5 receptors, we performed experiments in mGlu5 receptor knockout mice (mGlu5^-/-^) and their wild-type littermates (mGlu5^+/+^). All mice were obtained by heterozygous breeding. As expected, treatment with VU0360172 (30 mg/kg) significantly increased the InsP formation in the hippocampus, prefrontal cortex and striatum of mGlu5^+/+^ mice. In contrast, treatment with VU0360172 failed to stimulate PI hydrolysis in mGlu5^-/-^ mice (**Figure 3**). In mGlu5^-/-^ mice treated with vehicle, InsP levels were significantly reduced in the striatum and showed a trend to reduction in the prefrontal cortex and hippocampus, as compared to their wild-type counterparts (**Figure 3**). This suggests that endogenous activation of mGlu5 receptors contributes to basal PI hydrolysis in the mouse brain.

## Discussion

We have shown for the first time that it is possible to measure mGlu5 receptor-mediated PI hydrolysis in animals in response to a PAM with a high specificity and a high level of reproducibility. This represents a step forward with respect to conventional measurements of mGlu5 receptor signaling radioactive inositol. The method allows measurements of endogenous InsP levels and optimizes the use of animals without the need of pooling brain tissue. This method might be particularly appropriate for the assessment of mGlu5 receptor signaling in a variety of physiological and pathological conditions, and for the study of the action of different chemotypes of PAMs on native mGlu5 receptors. We find particularly attractive the possibility of directly analyzing mGlu5 receptor signaling in response to different classes of PAMs in relation to brain activation, as occurs during the execution of cognitive tasks. This information can be particularly valuable from a clinical standpoint considering that mGlu5 PAMs are under development for the treatment of schizophrenia (Foster and Conn, 2017; Maksymetz et al., 2017).

We were intrigued from the finding that basal PI hydrolysis (i.e., endogenous InsP levels determined in control mice) was not uniform in different brain regions, and was higher in the prefrontal cortex regardless of the strain of mice. Endogenous activation or constitutive activity of mGlu5 receptors accounted for the high basal InsP levels in the prefrontal cortex, because levels were largely reduced by MTEP or by genetic deletion of mGlu5 receptors. The functional partnership between NMDA and mGlu5 receptors might explain the high endogenous activity of mGlu5 receptors in the prefrontal cortex. NMDA and mGlu5 receptors are physically linked by a chain of scaffolding proteins, which include PSD-95, Shank, and Homer (Tu et al., 1999). On the one side, activation of mGlu5 receptors facilitates NMDA receptor function by relieving the Mg^2+^ blockade of the NMDA-gated ion channel (Doherty et al., 1997; Ugolini et al., 1997; Tu et al., 1999; Awad et al., 2000; Attucci et al., 2001; Mannaioni et al., 2001; Pisani et al., 2001); on the other side, activation of NMDA receptors facilitates mGlu5 receptor activity by limiting mGlu5 receptor desensitization as a result of protein phosphatase-2B-mediated receptor dephosphorylation (Alagarsamy et al., 2005). At least in one population of prefrontal cortical GABAergic interneurons, the fast-spiking parvalbumin-positive interneurons, NMDA receptors are highly expressed and are tonically active because of the more depolarized state of these neurons (Homayoun and Moghaddam, 2007; Moghaddam and Javitt, 2012; Rosenthal-Simons et al., 2013). mGlu5 receptors are also present in parvalbumin-positive interneurons and parvalbumin-cell ablation of either NMDA or mGlu5 receptors alters the development and function of these neurons inducing core features of schizophrenia and other neurodevelopmental disorders (Belforte et al., 2010; Billingslea et al., 2014; Barnes et al., 2015). It is possible that the tonic activity of NMDA receptors in prefrontal cortical interneurons enhances the endogenous activation of mGlu5 receptors, accounting for the high mGlu5-dependent basal PI hydrolysis we have seen in the mouse prefrontal cortex. It will be interesting to examine how basal prefrontal cortical PI hydrolysis changes after conditional ablation of NMDA receptors in GABAergic interneurons or in response to treatments with NMDA receptor blockers, such as ketamine or phencyclidine.

To pharmacologically activate mGlu5 receptors we used the compound VU0360172 which displays high potency (EC_50_ value in the low nanomolar range) and selectivity as mGlu5 receptor PAM (Rodriguez et al., 2010). VU0360172 interacts with the FBEP/MPEP allosteric site of mGlu5 receptors (Rook et al., 2015), and behaves as a PAM with low agonist activity (Sengmany et al., 2017). VU0360172 stimulated PI hydrolysis at the dose of 30 mg/kg, but not at lower doses, in agreement with the dose response effect of the drug in behavioral studies (Rodriguez et al., 2010). VU0360172 stimulated PI hydrolysis to a different extent in the six selected brain regions, with the greatest PI response being observed in the hippocampus and the lowest response in the cerebellum. In CD1 mice, this roughly correlated with the expression levels of mGlu5 receptors, taking into account that the response to VU0360172 in the prefrontal cortex might be underestimated because of the high level of endogenous activation of mGlu5 receptors. We were surprised to find that the mGlu5 NAM MTEP was unable to antagonize the stimulation of InsP formation by VU0360172 in the prefrontal cortex whereas it was effective in the other brain regions. Perhaps in the prefrontal cortex mGlu5 receptors are highly activated by a mechanism of receptor-receptor interaction with NMDA receptors, and, under these conditions, receptors affinity for VU0360172 increases and the drug is no longer displaced by MTEP (at least at the doses used in the present study). It will be interesting to examine whether both basal and VU0360172-stimulated PI hydrolysis in the prefrontal cortex are affected by NMDA receptor blockers. The PI response to VU0360172 in CD1 mice was greater in the striatum than in the hypothalamus and olfactory bulb in spite of the comparable expression levels of mGlu5 receptors in the three regions. This might reflect differences in the efficiency of receptor coupling or in the physiological pattern of receptor activation.

## Conclusion

This new method for non-radioactive, *in vivo* assessment of mGlu5 receptor-mediated PI hydrolysis might offer opportunity for the investigation of how mGlu5 receptor signaling changes in relation to network activity and processes of activity-dependent synaptic plasticity, as well as in pathological conditions. Mouse models of monogenic autism are just an example of the multitude of potential applications of this method (see section “Introduction” and “References” therein). In addition, the method may be particularly advantageous for the screening of mGlu5 receptor PAMs in living animals, providing accurate information of how different classes of PAMs influence receptor function in different brain regions.

## Author Contributions

AZ, GA, GDC, LDM, LI, and RO performed experiments on *in vivo* PI hydrolysis and Western Blotting. AZ, GB, and LDM analyzed the data. AZ, GB, PC, and FN designed the research and wrote the manuscript.

## Conflict of Interest Statement

The authors declare that the research was conducted in the absence of any commercial or financial relationships that could be construed as a potential conflict of interest.
